# Saturated, Monounsaturated and Polyunsaturated Fatty Acids Intake and Risk of Pancreatic Cancer: Evidence from Observational Studies

**DOI:** 10.1371/journal.pone.0130870

**Published:** 2015-06-25

**Authors:** Xu Yao, Zhong Tian

**Affiliations:** Department of General Surgery, Shengjing Hospital, China Medical University, Shenyang, P. R. China; University of Florida, UNITED STATES

## Abstract

**Background:**

Although the relationship between dietary monounsaturated fatty acids (MUFAs), polyunsaturated fatty acids (PUFAs), and saturated fatty acids (SFAs) intake and pancreatic cancer risk has been reported by several studies, the evidence is controversial. We firstly conducted this comprehensive meta-analysis to summarize the aforementioned evidence from observational studies.

**Methods:**

The MEDLINE (PubMed), Embase, and ISI Web of Science databases were used to search for epidemiological studies of dietary SFA, MUFA, and PUFA and pancreatic cancer risk that were published until the end of June 2014. Random- or fixed-effects models were used to estimate the relative risks (RRs) and 95% confidence intervals (CIs). We also carried out subgroup, sensitivity, and publication bias analyses.

**Results:**

We identified 13 case-control studies and 7 prospective studies which including 6270 pancreatic cancer cases in the meta-analysis of SFA, MUFA, and PUFA and risk of pancreatic cancer. The summary RR was 1.13 (95%CI = 0.94-1.35, *I* ^2^ = 70.7%) for SFA, 1.00 (95%CI = 0.87-1.14, *I* ^2^ = 43.4%) for MUFA, and 0.87 (95%CI = 0.75-1.00, *I* ^2^ = 55.3%) for PUFA for high *versus* low intake categories. We found no evidence of publication bias.

**Conclusion:**

In summary, findings of this study supports an inverse association between diets high in PUFA and pancreatic cancer risk. Further large prospective studies are warranted to report the results stratified by the subtypes of MUFA and PUFA and adjust for other potential risk factors to eliminate residual confounding.

## Introduction

The cancer of pancreas is one of the most severe cancers, with approximately 0.3 million new cases diagnosed in 2012 all over the world, accounting for 2.4% of all cancer cases [1–2]. The prognosis of this disease is extremely poor with median survival time ≤6 months [2–4]. The etiology of this disease is not well known, but multiple risk factors, including cigarette smoking, diabetes mellitus, obesity, parity and genetic factors have been associated with pancreatic cancer risk [5–10]. Since early detection of this disease is still in an exploratory stage, an important way is to focus on prevention through identifying additional modifiable risk factors.

Given dietary factors may partly involved in the development of pancreatic cancer [11–12], understanding this potential role would bring more public health benefits. The Continuous Update Project for pancreatic cancer from the World Cancer Research Fund and the American Institute for Cancer Research (WCRF/AICR) concluded the relationship between fat intake and risk of pancreatic cancer as “limited-no conclusion” [11]. Meanwhile, the most recently published meta-analysis which included 6 prospective and 13 case-control studies found no evidence between that total fat consumption and pancreatic cancer risk [13], which was consistent with the aforementioned report of the WCRF/AICR. However, experimental studies have suggested that polyunsaturated fatty acidconcls (PUFAs), but not monounsaturated (MUFAs) or saturated fatty acids (SFAs), inhibit human pancreatic cancer cell growth, which indicate the relationship between fats consumption and risk of pancreatic cancer may rely on the level of specific fatty acids intake [14–15]. Besides, SFA promotes insulin resistance, whereas MUFA and PUFA improve insulin resistance [16], which might involved in pancreatic cancer development [16–17]. On the other hand, although the relationships between different fatty acids intake and pancreatic cancer risk have been researched extensively, the epidemiologic evidence remains inconsistent and elusive. To our knowledge, no comprehensive meta-analysis on this topic is available recently. Therefore, in order to further assess the role of different fatty acids on the risk of pancreatic cancer, we evaluated all published data from observational studies, using a meta-analytic method.

## Materials and Methods

### Search Strategy

We carried out this study following the reporting guidelines of Meta-analysis Of Observational Studies in Epidemiology (MOOSE) [18]. A literature search to the end of June 2014 was carried out using PubMed, EMBASE, and ISI Web of Science databases by these key words: (diet or dietary or fat or fatty) and (pancreatic or pancreas) and (cancer or neoplasm). Additionally, we searched the reference lists of retrieved articles for additional studies.

### Study Selection

**Fig 1:**
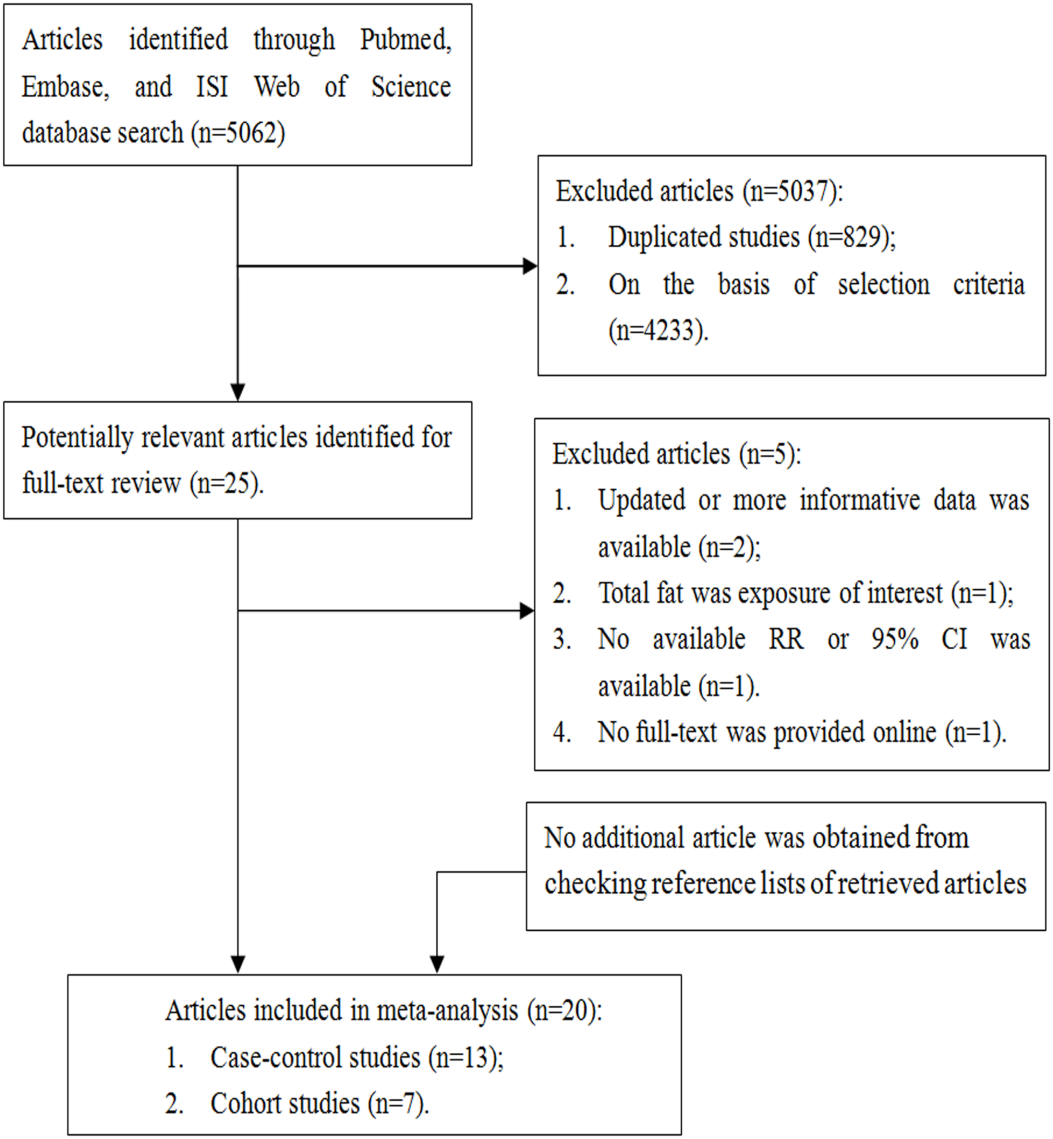
Flow-chart of study selection.

Two investigators (XY and ZT) independently evaluated the titles and abstracts of potentially studies using the following inclusion criteria: (1) the study had a cohort/case-cohort/nested case-control/case-control study design; (2) the exposure was dietary SFA, MUFA, or PUFA intake; (3) the outcome was the incidence of pancreatic cancer; and (4) provided relative risks (RRs), odds ratios (ORs), and hazard ratios (HRs) with 95% confidence intervals (CIs). If multiple articles were based on the same study population, the one with more informative data was selected. We identified 20 potentially relevant studies [12,15,17,19–35] from 5062 articles (Fig 1).

### Data Extraction

Two independent investigators (XY and ZT) evaluated the eligibility and abstracted the data of each study. The following information were extracted from included studies: the last name of first author, publication year, geographic location(s), study design, sample size of the study, individuals’ ages, prospective studies' follow-up years, exposure and outcome methods and SFA, MUFA, and PUFA intake categories, adjusted risk estimates and their 95% CIs of each study, and factors matched in the design or potential confounders adjusted for in the data analysis [36]. We abstracted the risk estimates which demonstrated the greatest degree of control for potential confounders from each included study.

### Quality assessment

Two independent investigators (XY and ZT) used a scoring system with 9-star on the strength of the Newcastle-Ottawa Scale (NOS) to evaluate the quality of included studies [36–37]. Three quality parameters: selection, comparability, and exposure/outcome evaluation were used to evaluate the observational studies. The full score is 9, with a score of 7 or higher indicating a high study quality in this study.

### Statistical analysis

The Higgins and Thompson fixed-effects model [38] was used if we observed non-significant heterogeneity. The DerSimonian and Laird random-effects model [39] was used if we observed significant heterogeneity. Galbraith plot was used to visual depict the heterogeneity. These two models were used to estimate summary RR and 95%CIs for the highest *versus* lowest categories of these interested exposures [8]. When evaluating heterogeneity among studies, we used the *I* ^2^ statistics [38]. Small study bias, such as publication bias, was assessed through Egger’s [40], Begg’s methods [41], and funnel plots. To find the source of heterogeneity, subgroup analyses were carried out by the following variables: study design (cohort *versus* case-control study), study quality (high *versus* low), geographic location (North America, Europe, and other), energy-adjusted models (yes *versus* no), validated food frequency questionnaires (FFQs) (yes *versus* no), and confounders that were adjusted for the following: cigarette smoking, body mass index, diabetes mellitus, and alcohol drinking. Finally, in order to provide a consistent approach to meta-analysis, we performed sensitivity analyses via ruling out each study alternately to reflect the influence of individual results on the overall estimate [13]. Statistical analyses were conducted with Stata software (Version 12.0; StataCorp). The log files of these analyses were available online (S1 Stata Log)

## Results

### Study characteristics and quality assessment

We identified 13 case-control studies including 3198 cases and 10,902 controls and 7 prospective studies involving 3072 cases and 1,130,815 individuals in this study. S1 Table summarizes the characteristics of these included studies which were conducted in the North America (n = 12), Europe (n = 6), and others (including Asia and Australia) (n = 2). Age and cigarette smoking were adjusted for all the included studies (n = 20). Energy intake (n = 17) and history of diabetes (n = 12) were adjusted for in the most studies. Body mass index (n = 8) and/or alcohol drinking (n = 5) were adjusted for in fewer studies.

S2 and S3 Tables demonstrated the quality scores of each included studies. The range of study-specific quality scores was from 6 to 9, and were 7 or greater for the majority (15 of 20) of included studies.

### Saturated Fatty Acids

**Fig 2:**
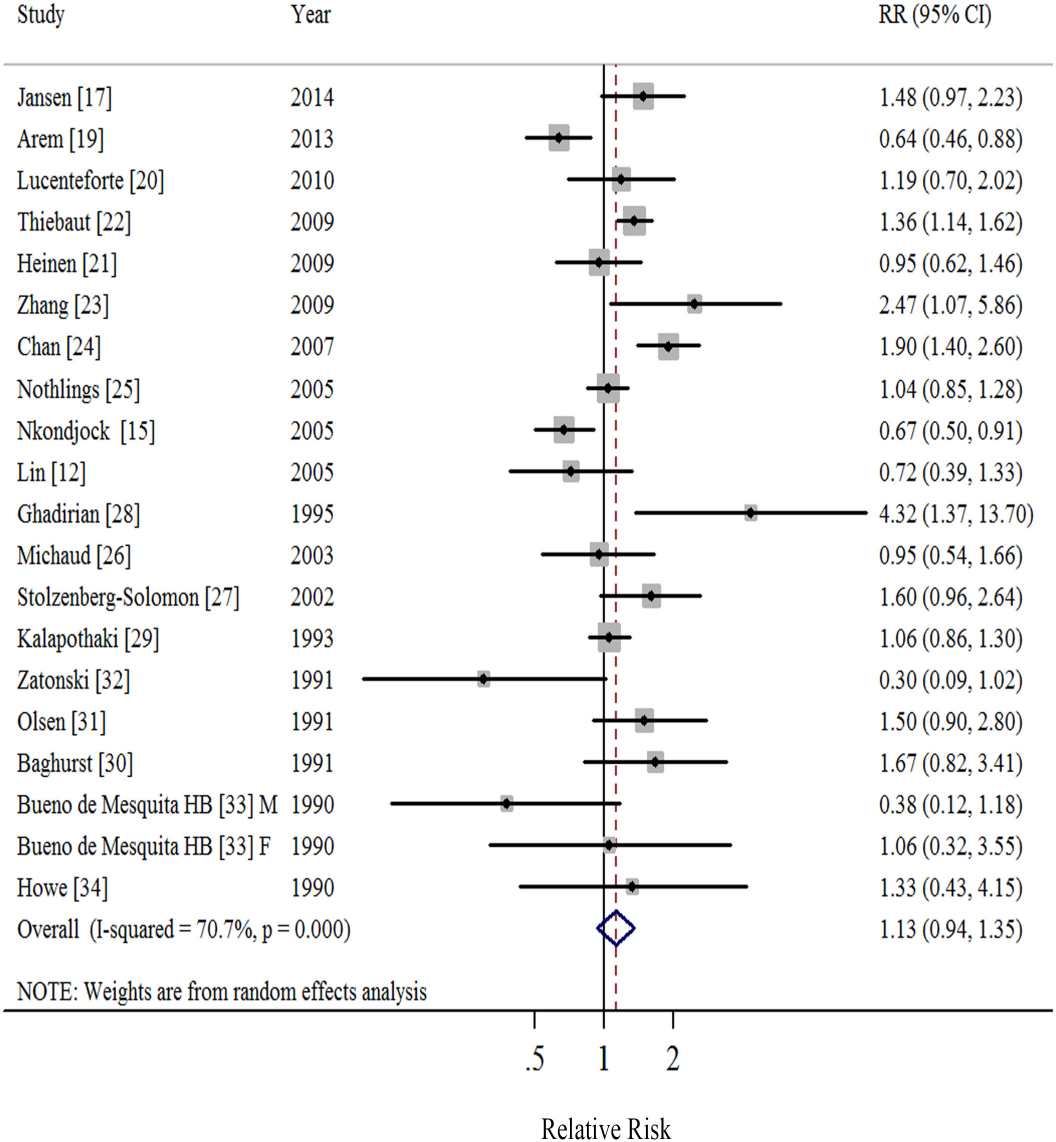
Forest plots (random effect model) of meta-analysis on the relationship between saturated fatty acids intake and pancreatic cancer risk. Squares indicate study-specific relative risks (size of the square reflects the study-specific statistical weight); horizontal lines indicate 95% CIs; diamond indicates the summary relative risk estimate with its 95% CI. M: male; F: female; RR: relative risk.

Nineteen studies [12,15,17,19–34] demonstrated results for high *versus* low intake of SFA and risk of pancreatic cancer. A random-effects model yielded a summary RR of 1.13 (95%CI = 0.94–1.35), with significant heterogeneity (*I* ^2^ = 70.7%, *P*<0.001; Table 1, Fig 2, S1 Fig). We found no evidence of publication bias by the Egger’s (*P* = 0.976) and Begg’s method (*P* = 0.974) as well as in funnel plots when inspected visually. The RRs ranged from 1.08 (95%CI = 0.90–1.29, *I* ^2^ = 66.0%) when ruling out the study by Chan et al [24] to 1.18 (95%CI = 0.99–1.40, *I* ^2^ = 65.7%) when ruling out the study by Arem et al [19] in the sensitivity analysis.

**Table 1:** Summary risk estimates of the association between saturated, monounsaturated and polyunsaturated fatty acid intakes and pancreatic cancer risk.

	SFA				MUFA				PUFA			
	No.	Summary RR	*I* ^2^	*P* *	No.	Summary RR	*I* ^2^	*P* *	No.	Summary RR	*I* ^2^	*P* *
		95% CI	(%)	value		95% CI	(%)	value		95% CI	(%)	value
**Overall**	19	1.13 (0.94–1.35)	70.7	<0.0001	17	1.00 (0.87–1.14)	43.4	0.026	18	0.87 (0.75–1.00)	55.3	0.002
**Study Design**												
Cohort stu dies	6	1.04 (0.81–1.35)	74.2	0.002	5	1.07 (0.94–1.23)	37.0	0.175	6	0.94 (0.83–1.07)	7.0	0.372
Case-control studies	13	1.19 (0.90–1.56)	71.3	<0.0001	12	0.99 (0.81–1.21)	47.7	0.028	12	0.82 (0.65–1.02)	65.4	0.001
**Study Quality**												
High	14	1.13 (0.94–1.36)	74.2	<0.0001	13	1.02 (0.89–1.16)	40.5	0.064	14	0.90 (0.78–1.04)	52.5	0.011
Low	5	1.06 (0.50–2.23)	65.6	0.013	4	0.67 (0.31–1.46)	55.3	0.062	4	0.66 (0.38–1.15)	61.7	0.034
**Geographic Location**												
North A merica	11	1.23 (0.95–1.59)	80.2	<0.0001	9	1.05 (0.87–1.27)	52.7	0.031	10	0.86 (0.72–1.04)	58.5	0.010
Europe	6	1.05 (0.90–1.24)	41.3	0.116	6	0.92 (0.79–1.07)	36.0	0.153	6	0.91 (0.69–1.20)	52.7	0.048
Others	2	1.07 (0.47–2.45)	67.5	0.079	2	1.17 (0.73–1.88)	0	0.466	2	0.66 (0.31–1.38)	61.9	0.105
**Energy-adjusted Models** ^†^												
Yes	13	1.15 (0.96–1.38)	66.8	<0.0001	12	1.07 (0.97–1.18)	10.3	0.342	12	0.88 (0.76–1.02)	47.7	0.028
No	6	1.13 (0.62–2.07)	74.6	0.001	5	0.79 (0.84–1.31)	61.1	0.036	6	0.87 (0.57–1.31)	69.7	0.006
**Validated FFQ**												
Yes	13	1.14 (0.92–1.41)	76.1	<0.0001	12	1.04 (0.89–1.21)	45.9	0.041	13	0.88 (0.76–1.02)	44.5	0.042
No	6	1.07 (0.67–1.71)	58.6	0.024	5	0.93 (0.78–1.11)	38.0	0.153	5	0.74 (0.46–1.16)	72.4	0.003
**Adjustment for confounders**												
**Body Mass Index**												
Yes	7	1.06 (0.77–1.47)	85.4	<0.0001	7	1.01 (0.83–1.23)	61.3	0.017	8	0.91 (0.82–1.01)	16.6	0.299
No	12	1.18 (0.94–1.47)	49.2	0.023	10	0.97 (0.84–1.12)	26.7	0.190	10	0.83 (0.61–1.13)	68.3	<0.0001
**Diabetes Mellitus**												
Yes	11	1.18 (0.99–1.41)	69.3	<0.0001	10	1.06 (0.96–1.17)	26.4	0.200	11	0.88 (0.76–1.02)	50.6	0.027
No	8	1.03 (0.64–1.66)	68.4	0.001	7	0.87 (0.58–1.32)	51.2	0.045	7	0.85 (0.58–1.24)	64.0	0.007
**Alcohol Drinking**												
Yes	4	1.61 (1.21–2.14)	0	0.753	4	1.34 (1.00–1.79)	0	0.617	5	0.68 (0.41–1.13)	74.4	0.004
No	15	1.03 (0.85–1.27)	73.8	<0.0001	13	0.94 (0.81–1.09)	47.1	0.026	13	0.97 (0.89–1.06)	15.5	0.284

FFQ: food frequency questionnaire.

* *P* for heterogeneity within each subgroup.

^†^ Energy-adjusted models including nutrient density model, nutrient residual model, and energy partition model.

When stratified by study design, the summary RRs were 1.04 (95%CI = 0.81–1.35; *I* ^2^ = 74.2%) in cohort studies and 1.19 (95%CI = 0.90–1.56; *I* ^2^ = 71.3%) in case-control studies (Table 1). Although we observed no statistically significant results when stratified by geographic locations, the point estimate of studies conducted in North America (RR = 1.23) was slightly stronger than these in Europe (RR = 1.05) and other countries (RR = 1.07). In addition, when stratified by whether adjustment for potential confounders, significant positive associations were observed among those studies adjusted for diabetes mellitus or alcohol drinking (Table 1).

### Monounsaturated Fatty Acids

**Fig 3:**
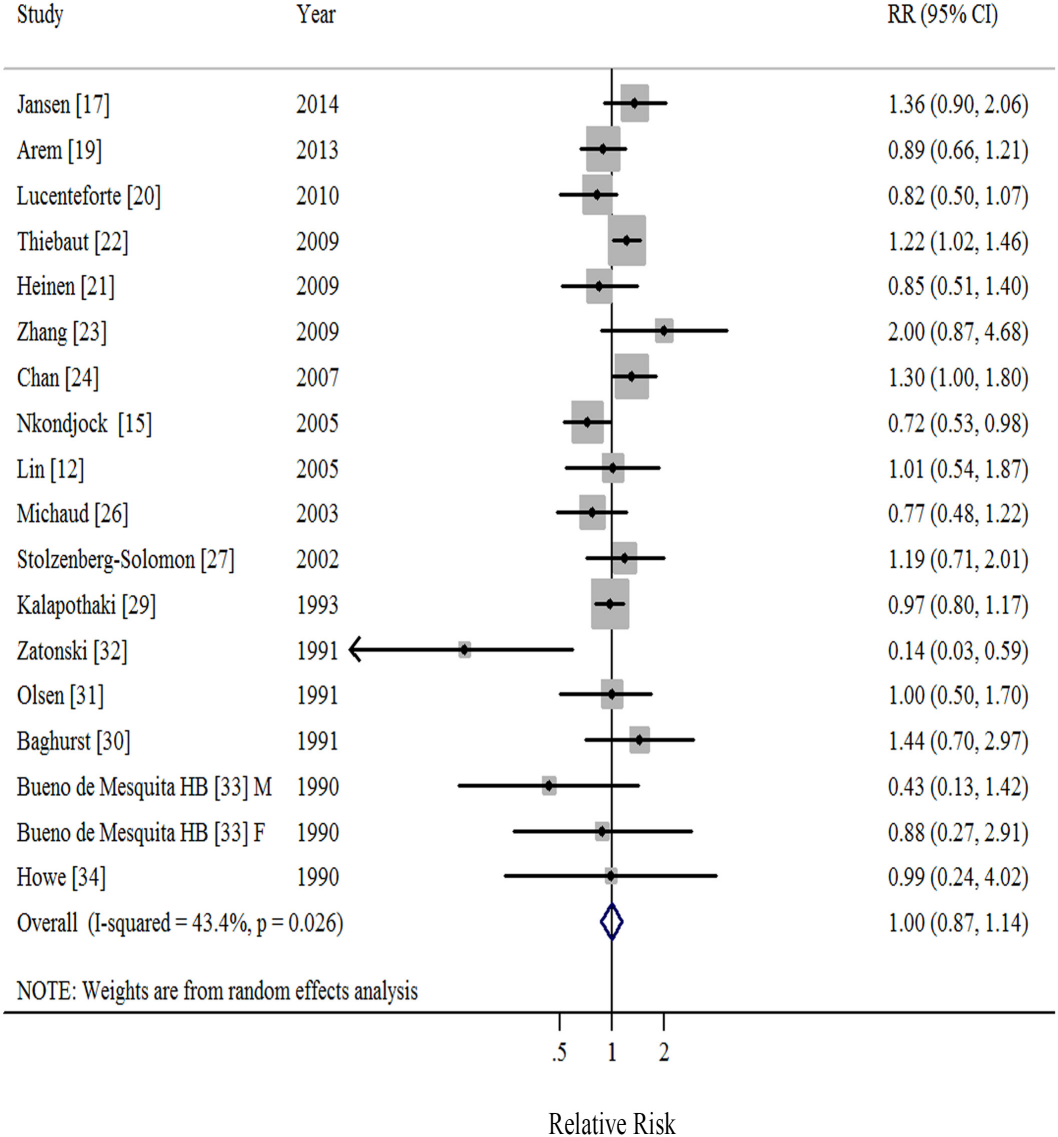
Forest plots (random effect model) of meta-analysis on the relationship between monounsaturated fatty acids intake and pancreatic cancer risk. Squares indicate study-specific relative risks (size of the square reflects the study-specific statistical weight); horizontal lines indicate 95% CIs; diamond indicates the summary relative risk estimate with its 95% CI. M: male; F: female; RR: relative risk.

Seventeen studies [12,15,17,19–24,26–27,29–34] demonstrated results for high *versus* low intake of MUFA and risk of pancreatic cancer. A random-effects model yielded a summary RR of 1.00 (95%CI = 0.87–1.14), with moderate heterogeneity (*I* ^2^ = 43.4%, *P* = 0.026; Table 1, Fig 3, S2 Fig). We found no evidence of publication bias by the Egger’s (*P* = 0.278) and Begg’s method (*P* = 0.449) as well as in funnel plots when inspected visually. The RRs ranged from 0.97 (95%CI = 0.84–1.12, *I* ^2^ = 36.4%) when ruling out the study by Nkondjock et al [15] to 1.03 (95%CI = 0.90–1.18, *I* ^2^ = 34.7%) when ruling out the study by Thiebaut et al [22] in the sensitivity analysis.

When stratified by study design, the summary RRs were 1.07 (95%CI = 0.94–1.23; *I* ^2^ = 37.0%) in prospective studies and 0.99 (95%CI = 0.81–1.21; *I* ^2^ = 47.7%) in case-control studies (Table 1). When stratified by whether adjustment for potential confounders, we observed significant positive association among those studies adjusted for alcohol drinking (Table 1).

### Polyunsaturated Fatty Acids

**Fig 4:**
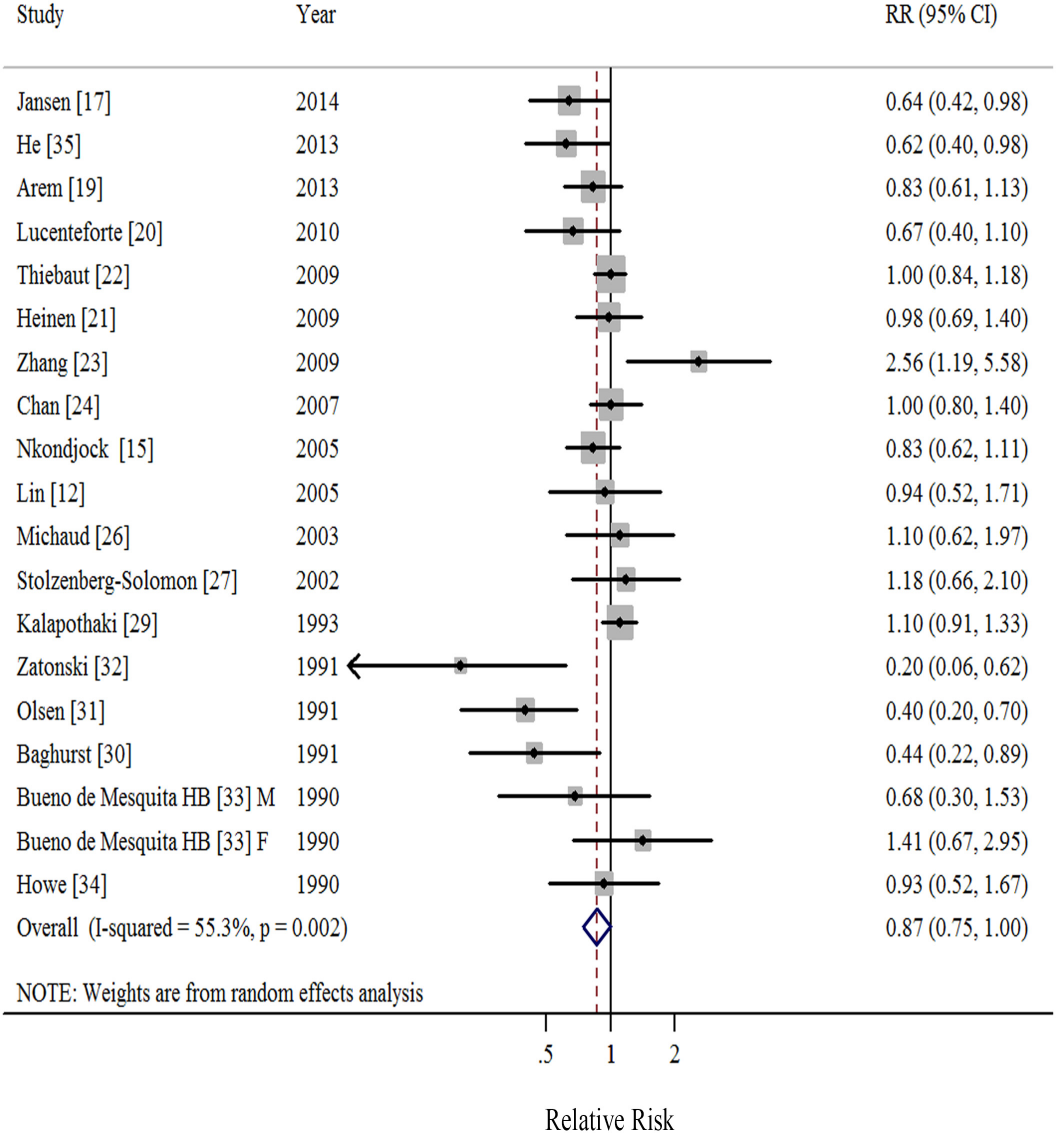
Forest plots (random effect model) of meta-analysis on the relationship between polyunsaturated fatty acids intake and pancreatic cancer risk. Squares indicate study-specific relative risks (size of the square reflects the study-specific statistical weight); horizontal lines indicate 95% CIs; diamond indicates the summary relative risk estimate with its 95% CI. M: male; F: female; RR: relative risk.

Eighteen studies [12,15,17,19–24,26–27,29–35] demonstrated results for high *versus* low intake of PUFA and risk of pancreatic cancer. A random-effects model yielded a summary RR of 0.87 (95%CI = 0.75–1.00), with moderate heterogeneity (*I* ^2^ = 55.3%, *P* = 0.002; Table 1, Fig 4, S3 Fig). We found no evidence of publication bias by the Egger’s (*P* = 0.097) and Begg’s method (*P* = 0.294) as well as in funnel plots when inspected visually. The RRs ranged from 0.84 (95%CI = 0.72–0.99, *I* ^2^ = 53.1%) when ruling out the study by Kalapothaki et al [29] to 0.90 (95%CI = 0.78–1.03, *I* ^2^ = 48.9%) when ruling out the study by Olsen et al [31] in the sensitivity analysis.

When stratified by study design, compared to the borderline significant result in case-control studies (RR = 0.82, 95%CI = 0.65–1.02; *I* ^2^ = 65.4%), we observed attenuated association in cohort studies (RR = 0.94, 95%CI = 0.83–1.07; *I* ^2^ = 7.0%). Similar borderline significant results were observed among studies with high quality, conducted in North America, studies using energy-adjusted models, and studies adjusted for body mass index, and diabetes mellitus (Table 1).

## Discussion

Finding from this meta-analysis comprising 20 epidemiological studies indicated that high intakes of PUFA were significant associated with a reduced pancreatic cancer risk as compared with low consumption. However, we found no statistically significant relationship between SFA and MUFA and pancreatic cancer risk. This meta-analysis, to our knowledge, firstly quantify the associations between different dietary fatty acids and pancreatic cancer risk.

The exact hypothesized biological mechanisms underlying the relationship between fatty acids consumption and pancreatic cancer risk remain speculative, yet several potential mechanisms partly explain the aforementioned association. Animal experiments suggested that fatty acids in chyme excited cholecystokinin releasing when entering the duodenum, which increasing the pancreatic susceptibility to carcinogens as well as causing the hyperplasia of acinar cell, therewith the pancreatic carcinomas development in rodents [42–43]. In addition, the more easily stored as energy but less efficiently oxidized for energy of SFA, which increasing the expression of genes associated with the proliferation of adipocyte [19,44]. A previous animal study addressed that rodents fed diets rich in SFA had greatest increase in pancreatic tumorigenesis [45]. Another possible explanation might related to insulin insensitivity or insulin resistance. Several studies have shown that SFA promote insulin resistance, whereas MUFA and PUFA improve insulin resistance [46], which might involved in pancreatic cancer development [16–17]. Furthermore, the binding and responsiveness of insulin was adversely alters by increasing the content of SFA or decreasing the content of PUFA through diet [47]. However, some studies have suggested that pancreatic cancer development is generally strengthened by long-chain ω-6 PUFA through accelerating prostaglandin synthesis [48–50], but inhibited by ω-3 PUFA through a reduction in the availability of prostaglandins [21,48,50]. Thus, further *in vivo* and *in vitro* studies should shed light on the underlying mechanisms between different FA intake and pancreatic cancer risk.

Pre-specified stratified analyses by study characteristics were performed to explore the sources of heterogeneity. When stratified by study design, heterogeneity for PUFA disappeared (*I* ^2^ = 0%) in cohort studies (Table 1). Although borderline significant inverse associations were observed in both subgroups, the risk estimates from case-control studies were far from the null than those from cohort studies (0.82 *versus* 0.94), which may reflect the influence of selection and recall biases in retrospective studies. In addition, since the high fatality of pancreatic cancer, the information of elected cases completing by proxy respondents in a portion of included studies [28,32–34], might bring about recall bias.

Compared with individual studies with relatively limited pancreatic cancer cases and study populations, this meta-analysis included almost 1.2 million participants with a total of 6270 pancreatic cancer cases, which would increase the statistical power to detect weaker associations. Limitations of our study also require consideration. First, we cannot control for confounders that were not adjusted for in the individual studies. A few studies adjusted for body mass index and alcohol drinking while the majority adjusted for age, cigarette smoking, and total energy intake, however, residual or unmeasured confounding cannot be excluded, which is always a concern in observational studies. Second, some degree of misclassification of fatty acids intake could prone to overestimation of the range of intake and underestimation of the magnitude of the association between dietary intake and risk of cancer [51–52]. Nonetheless, none of these included studies has provided risk estimates corrected for measurement errors. Besides, using a self-reported FFQ, 24-h recall or other dietary history questionnaire to assess the dietary intake rather than reflected by biological markers, though stratified analyses indicated that whether using validated FFQ did not significantly change the aforementioned associations (Table 1). Third, we observed significant heterogeneity in this meta-analysis, which may be related to the study design, different population groups, method of exposure measurement, and adjustment for potential confounders. In addition, varied methods were used by studies to report fatty acids intake and may lead to heterogeneity in the summary results [13]. On the one hand, some studies [17,19,22,25] analyzed the fatty acids intake according to densities, yet several studies [12,20–21,24,27,29–31] presented the residuals of the linear regression of fatty acids on energy. The other studies [15,23,26,28,32,34] just put fatty acids and total energy intake together in the multivariable models instead of utilizing aforementioned methods. However, the summary RRs were generally similar, no matter whether using energy-adjusted methods [53], and we found no heterogeneity when stratified by whether using the aforementioned methods. Finally, as such, the findings of this meta-analysis should only be interpreted as following: individuals consumed the most PUFA have a 13% lower risk of pancreatic cancer compared with those consumed the least. Because of different methods used to report fatty acids intake and limited data available among included studies, this meta-analysis failed to provide the information of dose-response analysis.

In summary, the current study suggests that diet high in PUFA is inversely associated with pancreatic cancer risk. This evidence was largely limited to case-control studies because the aforementioned inverse association was attenuated among prospective studies. Additionally, current results of this study are insufficient to support the relationship between dietary SFA and MUFA and pancreatic cancer risk. Further large prospective studies are warranted to report the results stratified by the subtypes of MUFA and PUFA and adjust for other potential risk factors to eliminate residual confounding.

## Supporting Information

S1 Database The database of the analysis between saturated, monounsaturated and polyunsaturated fatty acids intake and risk of pancreatic cancer. (XLS)

S1 Fig Galbraith plot corresponding to the relationship between saturated fatty acids intake and pancreatic cancer risk. (TIF)

S2 Fig Galbraith plot corresponding to the relationship between monounsaturated fatty acids intake and pancreatic cancer risk. (TIF)

S3 Fig Galbraith plot corresponding to the relationship between polyunsaturated fatty acids intake and pancreatic cancer risk. (TIF)

S1 PRISMA Checklist The PRISMA checklist for this meta-analysis. (DOC)

S1 Stata Log The Stata log files of the analysis between saturated, monounsaturated, polyunsaturated fatty acids intake and pancreatic cancer risk. (ZIP)

S1 Table Characteristics of studies included in the meta-analysis. (DOCX)

S2 Table Methodological quality of prospective studies included in the meta-analysis. (DOC)

S3 Table Methodological quality of case-control studies included in the meta-analysis. (DOC)
