# Supplementary figures and images for: Saturated, Monounsaturated and Polyunsaturated Fatty Acids Intake and Risk of Pancreatic Cancer: Evidence from Observational Studies
Source: PLoS One. 2015 Jun 25;10(6):e0130870. doi: 10.1371/journal.pone.0130870 (PMC4481405; doi:10.1371/journal.pone.0130870)

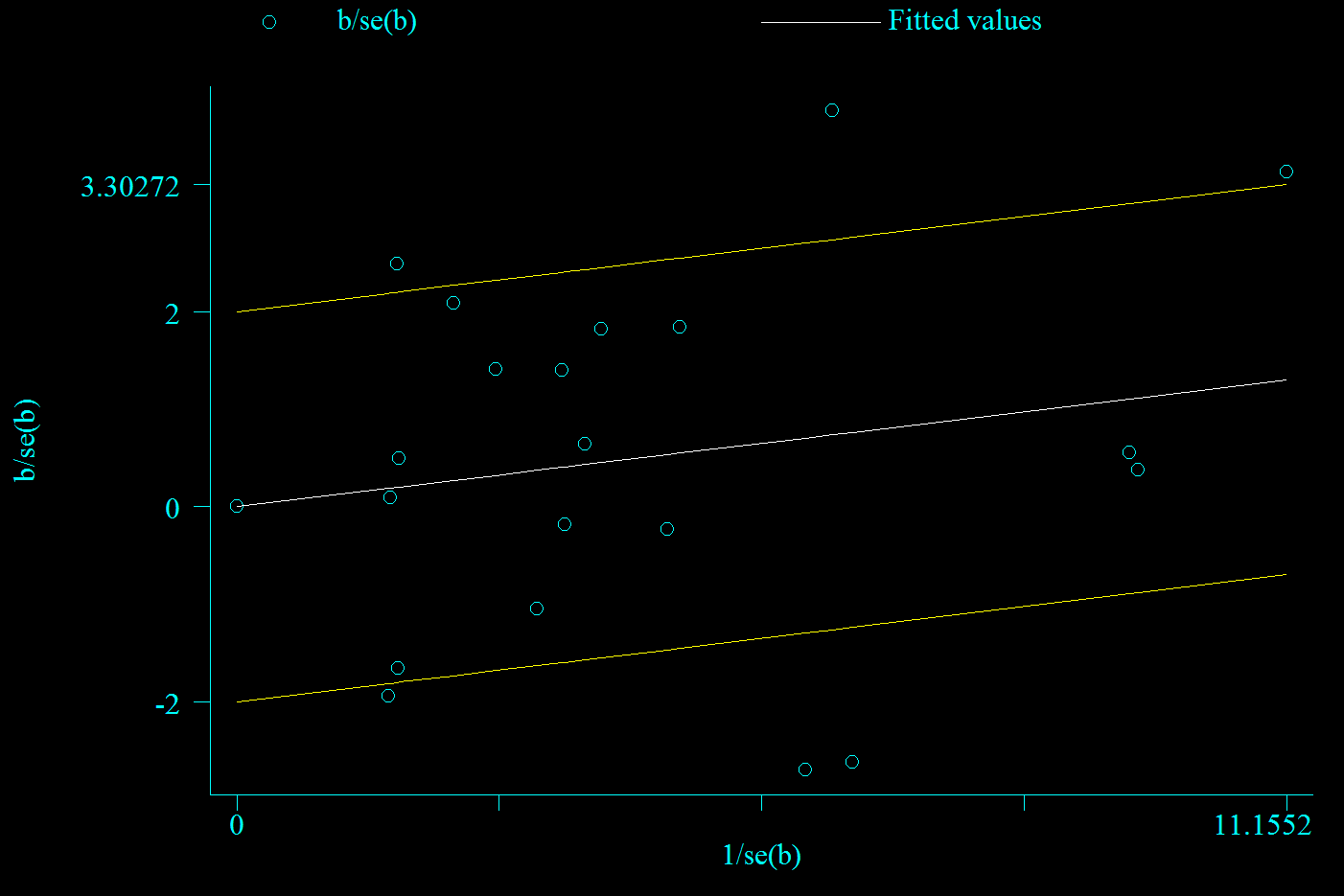

Supplement: S1 Fig — (TIF) [file pone.0130870.s002.tif]

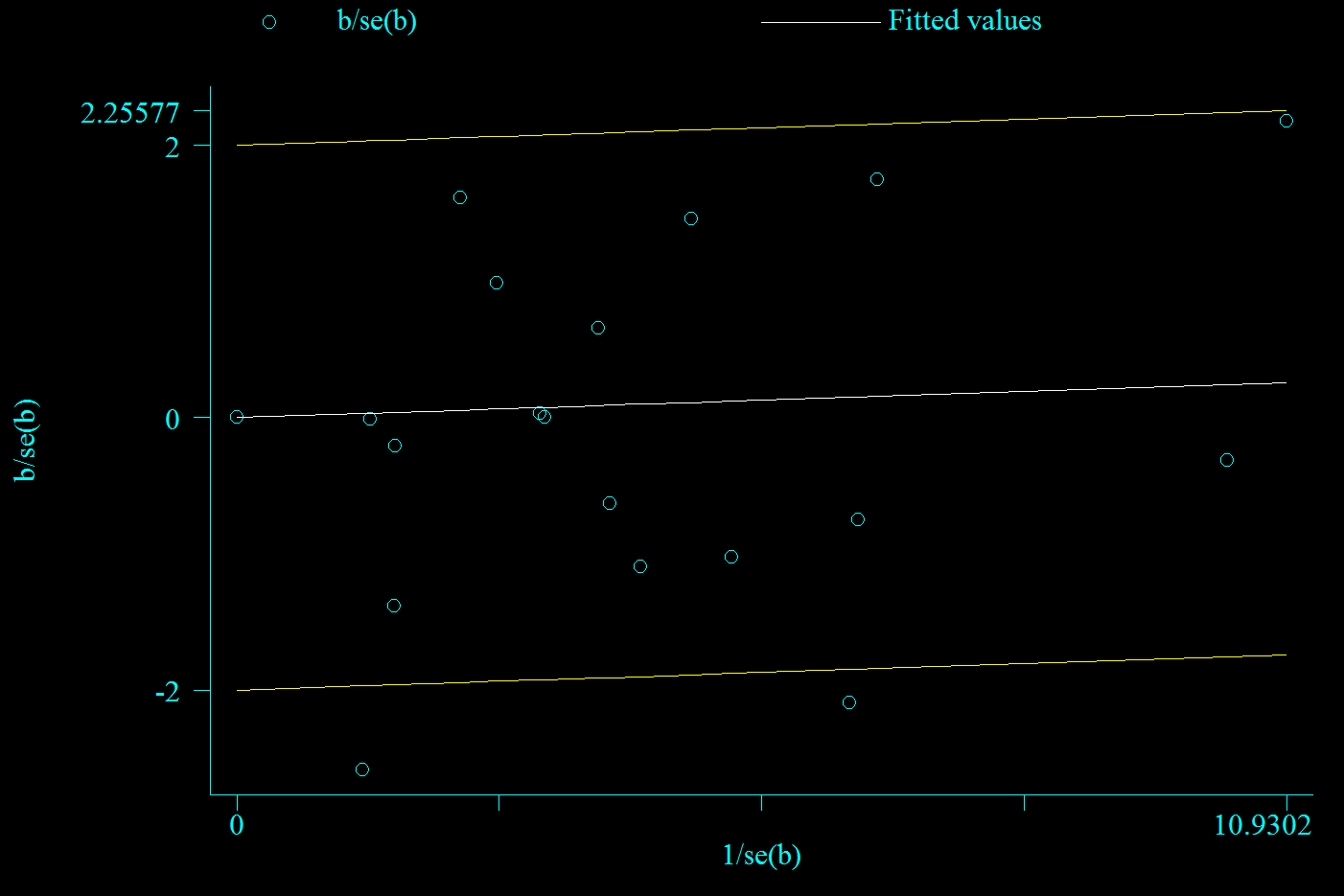

Supplement: S2 Fig — (TIF) [file pone.0130870.s003.tif]

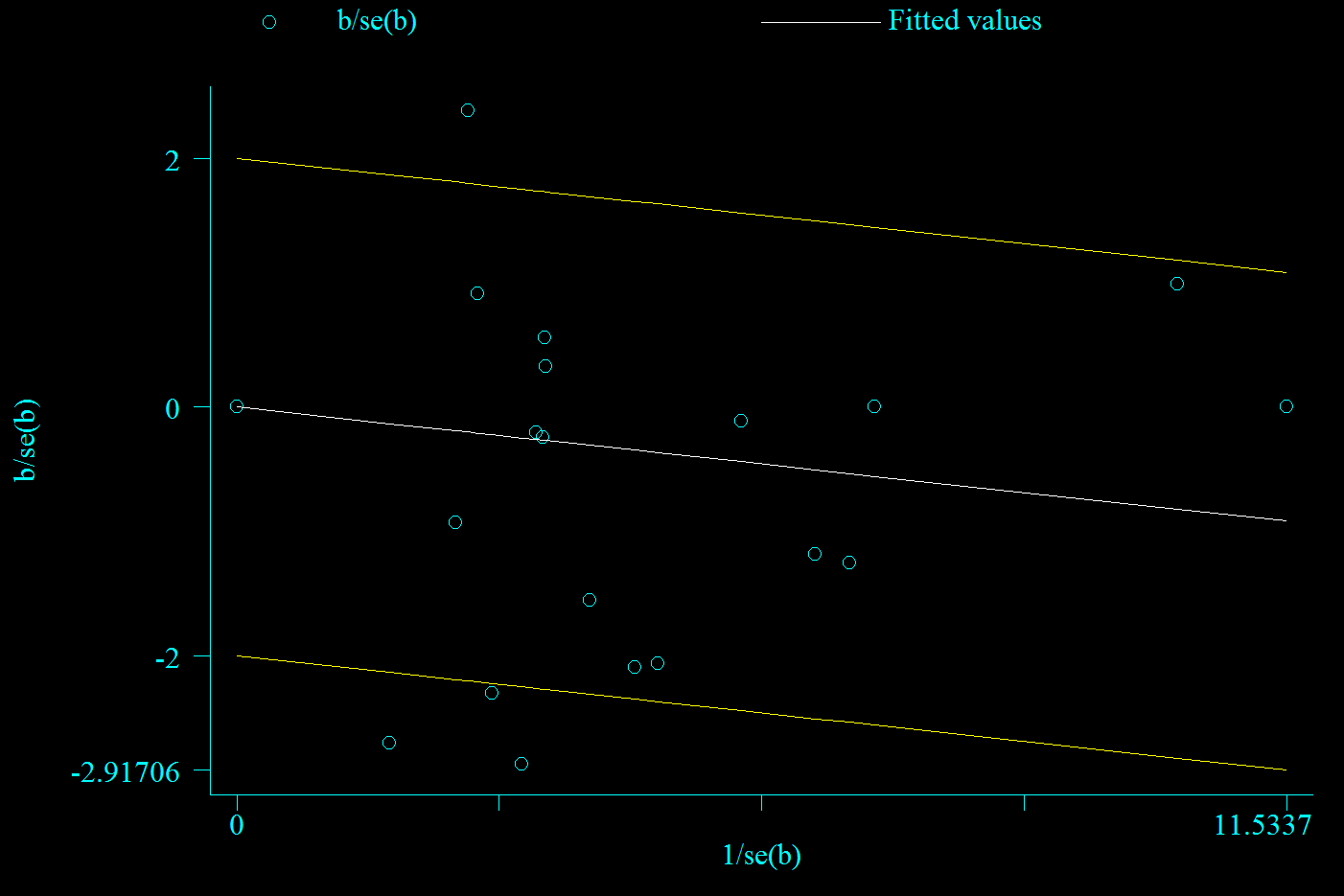

Supplement: S3 Fig — (TIF) [file pone.0130870.s004.tif]
