# Supplementary material for: Saturated, Monounsaturated and Polyunsaturated Fatty Acids Intake and Risk of Pancreatic Cancer: Evidence from Observational Studies
Source: PLoS One. 2015 Jun 25;10(6):e0130870. doi: 10.1371/journal.pone.0130870 (PMC4481405; doi:10.1371/journal.pone.0130870)
Supplement: S1 Table — (DOCX) [file pone.0130870.s007.docx]

**Table S1 Characteristics of studies included in the meta-analysis**

| **First author, year** | **Country** | **Sex** | **Study**  **period** | **No. of**  **cases** | **No. of controls**  **/size of cohort** | **Dietary**  **assessment** | **Matched/adjusted factors** |
| --- | --- | --- | --- | --- | --- | --- | --- |
| **Case-control study** |  |  |  |  |  |  |  |
| Jansen [17], 2014 | USA | M/F | 2004-2009 | 384 | 983 | Validated FFQ | Age, sex, cigarette smoking, usual adult BMI, DM, energy intake, number of drinks of alcohol per week and daily servings of total fruit and vegetable consumption |
| Lucenteforte [20], 2010 | Italy | M/F | 1991-2008 | 326 | 652 | Validated FFQ | Age, sex and center and year of interview, education, tobacco smoking, DM and total energy intake |
| Zhang [23], 2009 | USA | M/F | 1994-1998 | 186 | 554 | Validated FFQ | Age, sex, race, education, cigarette smoking, and alcohol intake, PA, fiber, vegetables and fruit intake |
| Chan [24], 2007 | USA | M/F | 1995-1999 | 532 | 1701 | Validated FFQ | Age, sex, BMI, race, education, smoking, and DM, energy intake [residual model] |
| Nkondjock [15], 2005 | Canada | M/F | 1994-1997 | 462 | 4721 | Validated FFQ | Age, province, educational attainment, smoking, BMI, total fat and energy intake |
| Lin [12], 2005 | Japan | M/F | 2000-2002 | 109 | 218 | Validated FFQ | Age, sex, energy intake and smoking |
| Ghadirian [28], 1995 | Canada | M/F | 1984-1988 | 179 | 239 | FFQ | Age, sex, lifetime cigarette consumption response status, and total energy intake |
| Kalapothaki [29], 1993 | Greece | M/F | 1991-1992 | 181 | 181 | FFQ | Age, gender, hospital, past residence, years of schooling, cigarette smoking, DM, energy intake |
| Zatonski [32], 1991 | Poland | M/F | 1985-1988 | 110 | 195 | DHQ | Age, sex, area of residence, cigarette lifetime consumption and other sources of calories |
| Olsen [31], 1991 | USA | M | 1980-1983 | 212 | 220 | FFQ | Age, total energy, cigarette usage, alcohol consumption, DM, and educational level |
| Baghurst [30], 1991 | Australia | M/F | 1984-1987 | 104 | 253 | Validated FFQ | Age, sex, total energy and for alcohol and tobacco usage |
|  |  |  |  |  |  |  | ***(Continued)*** |

**Table S1 (Continued)**

| **First author, year** | **Country** | **Sex** | **Study**  **period** | **No. of**  **cases** | **No. of controls**  **/size of cohort** | **Dietary**  **assessment** | **Matched/adjusted factors** |
| --- | --- | --- | --- | --- | --- | --- | --- |
| Bueno de Mesquita HB [33], 1990 | Netherlands | M/F | 1984-1988 | 164 | 480 | FFQ | Age, total smoking and response status |
| Howe [34], 1990 | Canada | M/F | 1983-1986 | 249 | 505 | DHQ | Age, sex, proxy status, smoking, protein, carbohydrate and fiber intake |
| **Cohort study** |  |  |  |  |  |  |  |
| He [35], 2013 | USA | M/F | 2000-2008 | 151 | 66,616 | Validated FFQ | Age, gender, ethnicity, education, BMI, PA, smoking status, alcohol consumption, diabetes mellitus, family history of pancreatic cancer, NSAID use, dietary intakes of fruits, vegetables, dairy products, red/processed meat, and calories |
| Arem [19], 2013 | USA | M/F | 1993-2009 | 411 | 111,416 | Validated DHQ | Age, gender, calories, DM, BMI, and smoking status |
| Thiebaut [22], 2009 | USA | M/F | 1995-2003 | 1337 | 525,473 | Validated FFQ | Age, sex, total energy intake, smoking history, BMI, and DM |
| Heinen [21], 2009 | Netherlands | M/F | 1986-1999 | 350 | 120,852 | Validated FFQ | Age, gender, energy, smoking, DM, history of hypertension, BMI, and vegetables and fruit intake |
| Nothlings [25], 2005 | USA | M/F | 1993-2001 | 482 | 190,545 | Validated FFQ | Age, sex, time on study, ethnicity, DM, familial history of pancreatic cancer, smoking status, and energy intake |
| Michaud [26], 2003 | USA | F | 1976-1998 | 178 | 88,802 | Validated FFQ | Age, pack-years of smoking, BMI, DM, caloric intake, height, PA, menopausal status, and glycemic load intake |
| Stolzenberg-Solomon [27], 2002 | Finland | M | 1985-1997 | 163 | 27,111 | Validated FFQ | Age, smoking, ATBC trial interventions, dietary folate, carbohydrate intakes, history of diabetes mellitus, occupational physical activity, education and total energy |

ATBC, Alpha-Tocopherol, Beta-Carotene Cancer Prevention Study; BMI, body mass index; DHQ, dietary history questionnaire; DM, diabetes mellitus; F, female; FFQ, food frequency questionnaire; M, male; NSAID, non-steroidal anti-inflammatory drug; PA, physical activity.
