# Supplementary material for: Saturated, Monounsaturated and Polyunsaturated Fatty Acids Intake and Risk of Pancreatic Cancer: Evidence from Observational Studies
Source: PLoS One. 2015 Jun 25;10(6):e0130870. doi: 10.1371/journal.pone.0130870 (PMC4481405; doi:10.1371/journal.pone.0130870)
Supplement: S2 Table — (DOC) [file pone.0130870.s008.doc]

**Table S2. Methodological quality of prospective studies included in the meta-analysis^*^**

| **First author (reference), publication year** | **Representativeness of the exposed cohort** | **Selection of the unexposed cohort** | **Ascertainment of exposure** | **Outcome of interest not present at start of study** | **Control for important factor or additional factor^†^** | **Assessment of outcome** | **Follow-up long enough for outcomes to occur**^‡^ | **Adequacy of follow-up of cohorts^§^** | **Total quality scores** |
| --- | --- | --- | --- | --- | --- | --- | --- | --- | --- |
| He [35], 2013 | ⚝ | ⚝ | ⚝ | ⚝ | ⚝⚝ | ⚝ | — | ⚝ | 8 |
| Arem [19], 2013 | ⚝ | ⚝ | ⚝ | ⚝ | ⚝⚝ | ⚝ | ⚝ | ⚝ | 9 |
| Thiebaut [22], 2009 | ⚝ | ⚝ | ⚝ | ⚝ | ⚝⚝ | ⚝ | — | ⚝ | 8 |
| Heinen [21], 2009 | ⚝ | ⚝ | ⚝ | ⚝ | ⚝⚝ | ⚝ | ⚝ | ⚝ | 9 |
| Nothlings [25], 2005 | ⚝ | ⚝ | ⚝ | ⚝ | ⚝⚝ | ⚝ | — | ⚝ | 8 |
| Michaud [26], 2003 | ⚝ | ⚝ | ⚝ | ⚝ | ⚝⚝ | ⚝ | ⚝ | ⚝ | 9 |
| Stolzenberg-Solomon [27], 2002 | — | ⚝ | ⚝ | ⚝ | ⚝⚝ | ⚝ | — | ⚝ | 7 |

* A study could be awarded a maximum of one star for each item except for the item Control for important factor or additional factor.

† A maximum of 2 stars could be awarded for this item. Studies that controlled for cigarette smoking received one star, whereas studies that controlled for other important confounders such as body mass index, diabetes mellitus received an additional star.

‡ A cohort study with a follow-up time >7 y was assigned one star.

§ A cohort study with a follow-up rate >75% was assigned one star.
