# Supplementary material for: Saturated, Monounsaturated and Polyunsaturated Fatty Acids Intake and Risk of Pancreatic Cancer: Evidence from Observational Studies
Source: PLoS One. 2015 Jun 25;10(6):e0130870. doi: 10.1371/journal.pone.0130870 (PMC4481405; doi:10.1371/journal.pone.0130870)
Supplement: S3 Table — (DOC) [file pone.0130870.s009.doc]

**Table S3. Methodological quality of case-control studies included in the meta-analysis^*^**

| **First author [reference], publication year** | **Adequate definition of cases** | **Representativeness of cases** | **Selection of control subjects** | **Definition of control subjects** | **Control for important factor or additional factor†** | **Exposure assessment** | **Same method of ascertainment for all subjects** | **Non response Rate‡** | **Total quality**  **scores** |
| --- | --- | --- | --- | --- | --- | --- | --- | --- | --- |
| Jansen [17], 2014 | ⚝ | ⚝ | — | ⚝ | ⚝⚝ | ⚝ | ⚝ | — | 7 |
| Lucenteforte [20], 2010 | ⚝ | ⚝ | — | ⚝ | ⚝⚝ | ⚝ | ⚝ | ⚝ | 8 |
| Zhang [23], 2009 | ⚝ | ⚝ | ⚝ | ⚝ | ⚝ | ⚝ | ⚝ | ⚝ | 8 |
| Chan [24], 2007 | ⚝ | ⚝ | ⚝ | ⚝ | ⚝⚝ | ⚝ | ⚝ | ⚝ | 9 |
| Nkondjock [15], 2005 | ⚝ | ⚝ | ⚝ | ⚝ | ⚝⚝ | ⚝ | ⚝ | — | 8 |
| Lin [12], 2005 | ⚝ | ⚝ | ⚝ | ⚝ | ⚝ | ⚝ | ⚝ | — | 7 |
| Ghadirian [28], 1995 | ⚝ | ⚝ | ⚝ | ⚝ | ⚝ | — | ⚝ | — | 6 |
| Kalapothaki [29], 1993 | ⚝ | ⚝ | — | ⚝ | ⚝⚝ | — | ⚝ | ⚝ | 7 |
| Zatonski [32], 1991 | ⚝ | ⚝ | ⚝ | ⚝ | ⚝ | — | ⚝ | — | 6 |
| Olsen [31], 1991 | ⚝ | ⚝ | ⚝ | ⚝ | ⚝⚝ | — | ⚝ | — | 7 |
| Baghurst [30], 1991 | ⚝ | ⚝ | ⚝ | ⚝ | ⚝ | — | ⚝ | — | 6 |
| Bueno de Mesquita HB [33], 1990 | ⚝ | ⚝ | ⚝ | ⚝ | ⚝ | — | ⚝ | — | 6 |
| Howe [34], 1990 | ⚝ | ⚝ | ⚝ | ⚝ | ⚝ | — | ⚝ | — | 6 |

* A study could be awarded a maximum of one star for each item except for the item Control for important factor or additional factor.

† A maximum of 2 stars could be awarded for this item. Studies that controlled for cigarette smoking received one star, whereas studies that controlled for other important confounders such as body mass index, diabetes mellitus received an additional star.

‡ One star was assigned if there was no significant difference in the response rate between control subjects and cases by using the chi-square test (P＞0.05).
